# Prevalence, subtypes and risk factors of *Blastocystis* spp. infection among pre- and perimenopausal women

**DOI:** 10.1186/s12879-021-06815-z

**Published:** 2021-11-01

**Authors:** Danuta Kosik-Bogacka, Małgorzata Lepczyńska, Karolina Kot, Małgorzata Szkup, Natalia Łanocha-Arendarczyk, Ewa Dzika, Elżbieta Grochans

**Affiliations:** 1 Independent Laboratory of Pharmaceutical Botany, Department of Biology and Medical Parasitology, Pomeranian Medical University in Szczecin, Powstanców Wielkopolskich 72, 70-111 Szczecin, Poland; 2 Department of Medical Biology, School of Public Health, University of Warmia and Mazury, Żołnierska 14 C, 10-561, Olsztyn, Poland; 3 Department of Biology and Medical Parasitology, Pomeranian Medical University in Szczecin, Powstanców Wielkopolskich 72, 70-111 Szczecin, Poland; 4 Department of Nursing, Pomeranian Medical University in Szczecin, Żołnierska 48, 71-210 Szczecin, Poland

**Keywords:** *Blastocystis* subtypes, Socio-demographic and epidemiological factors, Hematological profile, Pre- and perimenopausal women

## Abstract

**Background:**

*Blastocystis* spp. are considered pathogenic or commensal organisms, although the majority of researchers suggest that these are neglected pathogens. The main aim of this study was to determine the prevalence and subtype distribution of *Blastocystis* spp. in pre- and perimenopausal women, with respect to socio-demographic (age and place of residence), and epidemiological factors, as well as drinking tap water, contact with domestic animals, traveling abroad, health status, and presence of gastrointestinal symptoms. Additionally, the objective was to compare hematological and biochemical parameters of *Blastocystis* spp. infected and uninfected women.

**Methods:**

The study included 425 women aged 45–60. Their stool samples were examined microscopically and analyzed by a conventional polymerase chain reaction (cPCR).

**Results:**

*Blastocystis* spp. were detected in 6.1% of pre- and perimenopausal women. Molecular analysis of the stool samples identified seven *Blastocystis* subtypes (ST1–ST4, ST6, ST7, and ST9). *Blastocystis* subtypes 2 and 3 were the most prevalent. The presence of *Blastocystis* spp. was not significantly related to socio-demographic and epidemiological factors. There were also no significant associations between *Blastocystis* spp. and blood parameters, or gastrointestinal symptoms.

**Conclusion:**

This study complements the limited available data on the prevalence of *Blastocystis* spp. in pre- and perimenopausal women. It is also the first report showing the presence of *Blastocystis* subtype 9 in Poland.

## Background

*Blastocystis* spp. are enteric protozoa of humans and many animals, including mammals, birds, reptiles, amphibians, and insects [1–3]. *Blastocystis* spp. are considered pathogenic or commensal organisms, although the majority of researchers suggest that these are neglected pathogens [4]. The prevalence of *Blastocystis* spp. infection was of ranges from 22 to 56% in European countries [5–7], whereas in Asian and African countries the rate is 37% to 100% [8–10].

Some researchers suggest that the risk of *Blastocystis* spp. infection increases with age, as *Blastocystis* spp. have been found most frequently in patients aged between 30 and 60 years [11–13] Based on research conducted among ≥ 65-year-old nursing home residents, Arserim et al. [14] noticed that intestinal infections are a common problem in the elderly, with *Blastocystis* spp. being the most common pathogens detected in the stool. Infections in the elderly can be associated with atypical symptoms. The reason for this could be the weakening of cellular and humoral immunity, caused by the effect of aging on the gut microbiota. In addition, the risk of intestinal infections in the elderly is increased by the elevated prevalence of chronic diseases in this age group [14].

*Blastocystis* spp. are polymorphic parasites with multiple morphological forms including vacuolar, multivacuolar, avacuolar, granular, ameboid, and cyst [15, 16]. The cyst form is considered to represent the infectious stage; its transmission takes place via the fecal–oral route and can occur via contaminated water or food, human-to-human contact, or animal-to-human contact [1, 17, 18]. Studies using a rat model have demonstrated that the infective dose must contain at least 10 cysts [19].

*Blastocystis* spp. have been observed in Scottish and Malaysian sewage samples [20] and ready-to-eat package salads in Italy [21]. The description of two suspected waterborne outbreaks of blastocystosis [22, 23], and the chlorine insensitivity of *Blastocystis* spp. cysts implicate chlorinated drinking water as a potentially significant transmission route [24, 25].

Pathogenicity, mode of transmission, genetic diversity, host specificity, and treatment of *Blastocystis* spp. infections are still not well understood [26]. *Blastocystis* spp. have been observed in ulcers in the cecum, transverse colon, and rectum of an immunocompetent individual [27]. They can also obstruct the appendiceal lumen [28]. The pathogenicity of *Blastocysis* spp. probably depends on the duration (acute or chronic) and intensity of infection, host genetic factors, or *Blastocystis* subtypes [29, 30].

To date, 22 subtypes (ST1–ST22) of *Blastocystis* spp. have been identified, based on the sequence analysis of the small subunit ribosomal RNA gene, with 10 (ST1 to ST9 and ST12) reported in humans of which ST3 is the most commonly detected subtype [31].

*Blastocystis* spp. infection causes blastocystosis [32]. The common symptoms including chronic and acute diarrhea, abdominal pain, nausea, vomiting, anorexia, weight loss, and dermatological symptoms (e.g., rash, itching, and urticaria) [33, 34].

The *Blastocystis* spp. infection is usually diagnosed using direct-light microscopy, trichrome staining, or xenic in vitro cultures. Molecular diagnosis by polymerase chain reaction (PCR) is becoming more widely used for the detection of *Blastocystis* spp. This method is costly but more sensitive than the direct smear and xenic culture [35]. A recent study suggests that the direct fluorescent antibody method has a higher sensitivity than native-Lugol examination and is fast and practical compared to other methods [36].

The gut microbiota plays role in maintaining a mucosal barrier, colonization of pathogens, and contributing to immune homeostasis [37]. In general, postmenopausal women show reduced immune system resistance to encountered infectious pathogens, resulting in an increased risk of inflammation [38]. Zhao et al. [39] were observed alterations of gut microbiota in postmenopausal women. *Blastocystis* spp. play a role in the ecology of the gut microbiota through their interaction with other microbial components [40]. It has been found that subtype ST7 potentially leads to an imbalance in the intestinal microbiota [41, 42]. We hypothesized that in postmenopausal women, there is an increased risk of *Blastocystis* spp. infection and clinical symptoms. The main aim of this study was to determine the prevalence and subtype distribution of *Blastocystis* spp. in pre- and perimenopausal women, with respect to socio-demographic (age and place of residence), and epidemiological factors, as well as drinking tap water, contact with domestic animals, traveling abroad, health status, and presence of gastrointestinal symptoms. Additionally, the objective was to compare hematological and biochemical parameters of *Blastocystis* spp. infected and uninfected women.

## Methods

### Participants

The study included 425 women aged 45–60 (mean age 54.3 ± 4.2 years) from the West Pomeranian Voivodeship (Poland) who had volunteered after receiving information about the study from local papers and information posters in public places. A detailed description of the study sample was presented by Szkup et al. [43].

The criteria for inclusion in the study were: female aged between 45 and 60 years with no current cancerous, psychiatric, or inflammatory diseases, and explicit written consent to take part in the study. Patients who had taken antibiotics, anti-diarrhea compounds, barium, bismuth, or mineral oil (≤ 3 weeks) were excluded.

Each participant had to fill a questionnaire. The questionnaire was given to each participant to obtain socio-demographic (age and urban/rural residence), and epidemiological data, such as type of water supply, contact with domestic animals, place of spending holidays (Poland or abroad), health status, presence of gastrointestinal symptoms such as abdominal pain, diarrhea, abdominal cramps, nausea, bloating and constipation, and chronic diseases.

## Assessment

### Stool examination

The participants received stool sample containers and standard instructions on the proper and safe collection and preservation of the samples. Urine free stool samples were collected in a clean, dry, and leak-proof container.

The stool samples collected from all patients were examined macroscopically in terms of consistency, and presence of blood, and helminths. During the microscopic examination of the stools, approximately 2 g of stool sample was mixed with a drop of saline solution (0.9%) and Lugol’s iodine (diluted in 1:5 distilled water). The intensity of *Blastocystis* spp. infection using a × 40 magnification was determined using four levels of parasite load: (1) very low—single protozoans in the whole preparation, (2) low—single protozoans in almost every field of vision, (3) medium—5 to 10 protozoans in every field of vision, (4) high— > 10 protozoans in every field of vision. Taking into consideration that microscopy requires skilled personnel to recognize different morphological forms without information about the subtypes, the positive and unsure samples by this method were tested using the *CoproELISA Blastocystis* test (Savyon Diagnostics Ltd, Israel) to detect *Blastocytis* spp. antigens. The samples positive in ELISA and/or microscopic method were subjected to conventional PCR (cPCR).

### Molecular assays

For molecular analysis, the stool was stored in 70% ethanol. The samples were washed three times in phosphate-buffered saline (PBS) prior to DNA extraction. DNA extraction was performed using the Sherlock AX (A&A Biotechnology, Poland) kit to perform conventional cPCR according to the manufacturer’s instructions.

The method used for detecting *Blastocystis* spp. in the samples and subtype analysis involved cPCR with the primers RD5 (5′-ATCTGGTTGATCCTGCCAGT-3′), and BhRDr (3′-GAGCTTTTTAACTGCAACAACG-5′) amplifying a ~ 600-base pair (bp) fragment of the 1.8 kbp small subunit ribosomal RNA (SSU rRNA) gene [44]. This region of the SSU-rRNA gene has been shown to provide sufficient information for differentiating subtypes of *Blastocystis* [44]. 2 µL of extracted DNA was added to an amplification mixture containing 1 µl (10 µM) of primers and 12.5 μl of PCR Master Mix Plus (A&A Biotechnology, Poland). To each mixture, nuclease-free water was added to provide a final reaction volume of 25 μl. Sterile water was used as a negative control, and *Blastocystis* ST2 DNA, diluted to 1:100 obtained from the stool samples of colonized volunteers, was used as a positive control. The amplification profile consisted of 30 cycles of denaturation, annealing and extension at 94 °C, 59 °C and 72 °C respectively, with a final extension step of 2 min at 72 °C. PCR products (5 μl) were analyzed on 1.35% agarose gel using electrophoresis and visualized with UV light. Positive PCR products were purified using Clean-Up (A&A Biotechnology, Poland) according to the manufacturer’s recommendations.

Sequencing on the samples subject to cPCR was done once using Macrogen Humanizing Genomics Europe (Amsterdam, The Netherlands). Primers used in the experiment: RD5 (5′-ATCTGGTTGATCCTGCCAGT-3′), and BhRDr (3′-GAGCTTTTTAACTGCAACAACG-5′). Sequences were edited and assembled using Finch TV v 1.4 (Geospiza Inc., Seattle, WA, USA). The sequences were analyzed by the BLAST website (https://blast.ncbi.nlm.nih.gov/Blast.cgi?PAGE_TYPE=BlastSearch). STs and ST (SSU-rDNA) alleles were called using the sequence query facility in the *Blastocystis* Sequence Typing website available at http://pubmlst.org/Blastocystis/. Subtypes were identified by determining the exact match or closest similarity to all known *Blastocystis* subtypes according to the classification by Stensvold et al. [35].

## Laboratory parameters

The blood samples were taken from the antecubital vein on an empty stomach (at least 8 h from the last meal) from each patient between 7.00 and 9.30 in the morning, after 10 min of chair-seated rest. The blood was collected separately into vacutainer tubes (Sarstedt, Germany): one containing 1 G/L of K2 EDTA and the other for biochemical analysis of serum (7 ml), in compliance with the relevant regulations. White blood cells (WBC), red blood cells (RBC), hemoglobin (HGB), hematocrit (HCT), mean corpuscular volume (MCV), mean corpuscular hemoglobin concentration (MCHC), mean corpuscular hemoglobin (MCH), platelet (PLT), neutrophils (NEU), eosinophils (EOS), basophils, (BASO), lymphocytes (LYM), monocytes (MONO) and C-reactive protein (CRP) were recorded. Morphological blood analysis was performed at the Diagnostics Laboratory in the Independent Public Clinical Hospital no. 1 in Szczecin, Poland.

### Statistical analysis

In order to establish possible relationships between *Blastocystis* spp. infection and socio-demographic and epidemiological data, hematological and biochemical parameters were evaluated using the Independent Samples t-Test, the χ^2^ test, and Odds ratio (OR). The differences were seemed statistically significant at p < 0.05.

## Results

In the microscopic examination, 28 women from 425 were found as *Blastocystis* spp. positive and samples from three participants could not be clearly determined. The ELISA test on positive samples in the microscopy method revealed colonization of *Blastocystis* in all of the 31 samples of the examinees. In cPCR analysis, 31 women were tested positive, whereas 26 of the cPCR amplicons were successfully sequenced.

### Stool examination

In the microscopic examination of stool samples, we found only *Blastocystis* spp; no other intestinal parasites were found in the stool samples. The vacuolar forms of *Blastocystis* spp. were observed in 28 women. In the stool samples from three participants, similar cells to the vacuolar form of *Blastocystis* spp. were observed. Nevertheless, they were imprecise and finally identified as *Yarrowia lipolytica* and bacteria of *Citrobacter* genus in the molecular method. Two samples, which were surely identified as *Blastocystis* spp. in the microscopic examination, were identified as the fungal class *Saccharomyces* or other eukaryote cells using molecular analysis.

### Molecular subtyping of *Blastocystis* isolates

**Fig. 1:**
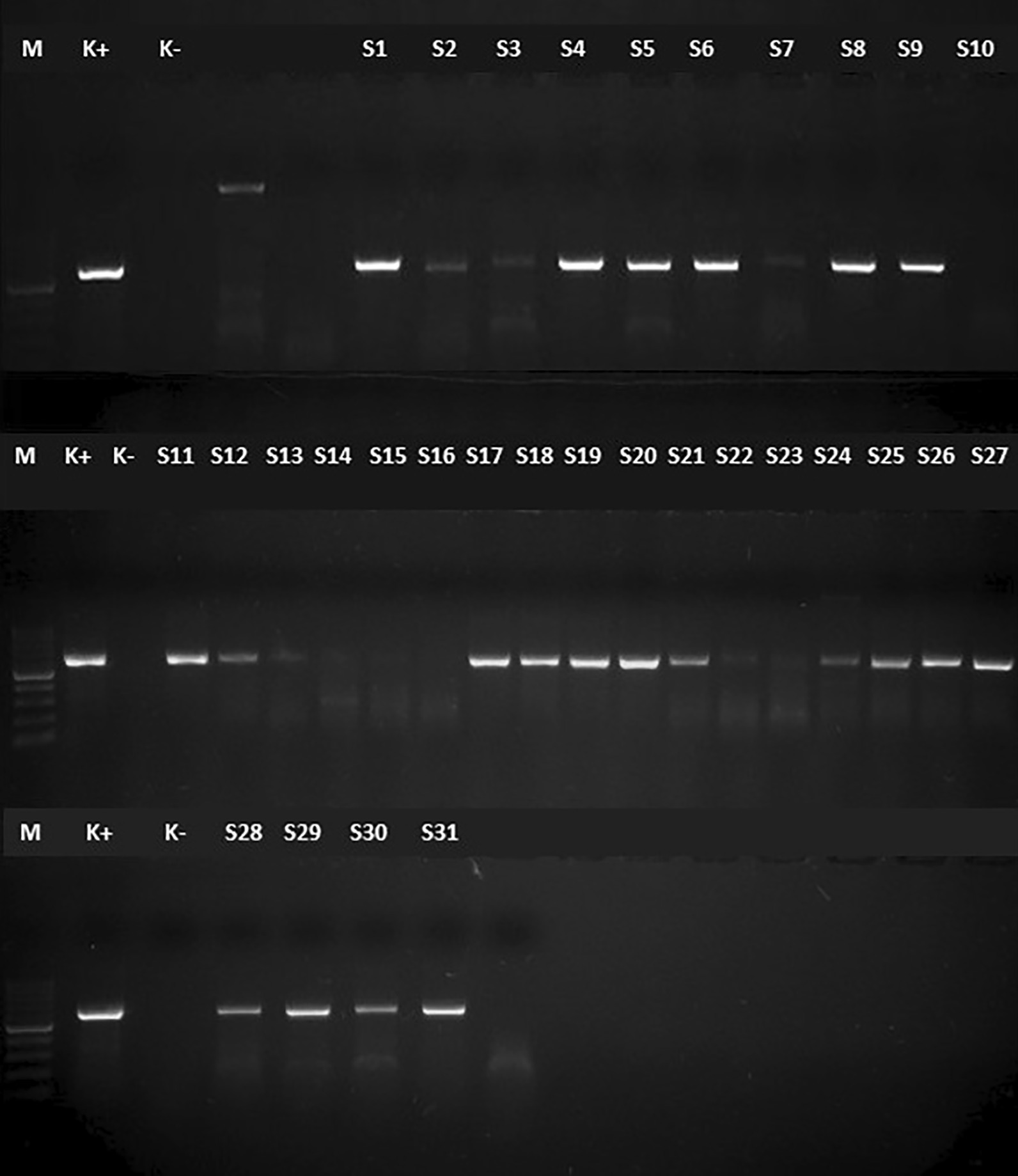
Electrophoresis result of the specific cPCR products of the stool samples of *Blastocystis* spp. positive samples in a 1.5% agarose gel. *Note* *PCR* polymerase chain reaction. M: 100 bp marker (cPCR: conventional polymerase chain reaction; K−: negative control; K+: Positive control; S1–S31: Patients' sample 600 bp)

By cPCR, 31 of the 425 women tested positive with the expected 600 bp fragment of the SSU-rDNA amplified, and 26 of the cPCR amplicons could be successfully sequenced (Fig. 1). The sequences from the remaining five samples indicated amplification of fungal DNA (the fungal class *Saccharomyces*, most probably *Yarrowia lipolytica*) as well as other eukaryote cells rather than *Blastocystis* spp.

The comparison between the representative sequences of *Blastocystis* subtypes, using the BLAST software, allowed the direct genotyping of the isolates according to the terminology by Stensvold et al. [35]. *Blastocystis* spp. were identified in 26 samples; seven *Blastocystis* subtypes (ST1–ST4, ST6, ST7, and ST9) were identified. ST2 and ST3 were the most prevalent subtypes, each found in 7/26 women followed by ST1 in 5/26, ST4 in 3/26. Two participants harbored ST6, and two more were colonized with ST7 and ST9 each.

Comparing diagnostic methods used in the current study it may be assumed that microscopy is one of the reliable methods to detect *Blastocystis* spp. in the stool samples. However, it should not be used as an ultimate method to identify protozoan parasites, but only as a screening of the samples.

### Socio-demographic and epidemiological characteristics of the women

A survey showed that of all women, 90.6% lived in urban areas and 9.4% in rural areas. 88.7% of respondents reported drinking tap water. Additionally, 96.0% of women declared having contact with domestic animals, including dogs, cats, guinea pigs, and hamsters (Table 1). We found an association between a place of spending holidays (in the country or traveling abroad) on *Blastocystis* spp. infection (p < 0.01); an odds ratio is less than 1 (OR 0.17) which means that there is a lower chance of *Blastocystis* spp. infection while traveling abroad than spending holidays in Poland. It is important to point out that not all women answer this question, and statistical analysis was done based on 182 patients.

**Table 1:** The risk factors of *Blastocystis* spp. infections (OR, odds ratio; p, level of significance; * due to lack of information from participants, statistics was done based on 182 patients)

Parameters	*Blastocystis* spp.		χ^2^ value	p	95% Confidence		p
	Positive	Negative			OR	Interval	
Residence-urban	22	363	0.73	0.39	2.37	0.31–18.02	0.41
Traveling abroad*	2	57	6.76	** < 0.01**	0.17	0.04–0.75	**0.02**
Tap water drinking	22	355	1.17	0.28	2.91	0.38–22.11	0.30
Eating unwashed fruits and vegetables	20	355	0.04	0.85	0.88	0.25–3.08	0.85
Eating undercooked meat	15	250	0.09	0.77	1.14	0.47–2.75	0.77
Contact with domestic animals	22	386	0.01	0.93	0.92	0.11–7.19	0.93
Not using gloves during gardening	6	102	0.01	0.94	0.96	0.37–2.51	0.94

Analyzing statistical data, it was also observed that women who lived in urban regions or drinking tap water have a bigger chance of getting infected with *Blastocystis* spp. than those patients who lived in rural areas and don’t drink tap water (OR > 1).

### Gastrointestinal symptoms

Patients marked in the questionnaires whether they had gastrointestinal symptoms that may suggest parasitosis. The women mostly complained about fatigue (64%), headache (53%), muscle pain (35%), and abdominal pain (26%). Table 2 presents all symptoms pointed by the participants. We found no statistically significant differences in symptoms between *Blastocystis* spp. infected and uninfected participants. However, in two parameters (fatigue and abdominal pain) level of significance was a little over 0.05, therefore it is recommended to check those parameters in the study conducted on a bigger population.

**Table 2:** The symptoms reported by *Blastocytis* spp. infected and uninfected patients (OR, odds ratio; p, level of significance)

Symptoms	*Blastocystis* spp.		χ^2^ value	p	95% Confidence		p
	Positive	Negative			OR	Interval	
Diarrhea	1	52	1.47	0.23	0.31	0.04–2.32	0.25
Nausea	1	47	1.17	0.28	0.34	0.05–2.61	0.30
Vomiting	1	7	0.80	0.37	2.56	0.30–21.76	0.39
Abdominal pain	10	102	3.67	0.06	2.26	0.96–5.31	0.06
Weight loss	1	23	0.08	0.78	0.75	0.10–5.81	0.78
Fatigue	19	253	3.65	0.06	2.80	0.93–8.38	0.07
Loss of appetite	1	21	0.03	0.85	0.82	0.11–6.42	0.85
Fever	1	17	0.00	0.98	1.03	0.13–8.09	0.98
Enlarged lymph nodes	1	20	0.02	0.89	0.87	0.11–6.77	0.89
Flu-like symptoms	2	87	2.20	0.14	0.34	0.08–1.50	0.16
Headache	11	213	0.23	0.63	0.81	0.35–1.89	0.63
Muscle pain	12	138	3.03	0.08	2.09	0.90–4.85	0.09

It was noted that people who have gastrointestinal symptoms, such as vomiting, abdominal pain, fatigue, and muscle pain, have a bigger chance of being infected with *Blastocystis* spp. than those women without those symptoms (OR > 1).

### Biochemical and haematological parameters in the blood

The mean values for hematological and biochemical were within normal limits (Table 3). Fifteen women with low hemoglobin and hematocrit levels were defined as anemic, including one woman infected with *Blastocystis* ST3. The participant had all parameters lower in the red blood cell’s profile that indicate microcytic anemia. Taking into account white blood cell’s profile, all *Blastocystis* spp. infected women had blood parameters in terms of reference values. There was no difference in hematological and biochemical parameters between *Blastocystis* spp. infected and uninfected participants. Among *Blastocystis* spp. infected patients, only one woman who was infected with ST2 had a higher level of CRP (8.38 mg/l).

**Table 3:** The hematological and biochemical profile of *Blastocystis* spp. infected and uninfected patients (*WBC* white blood cells; *RBC* red blood cells; *HGB* hemoglobin; *HCT* hematocrit; *MCV* mean corpuscular volume; *MCH* mean corpuscular hemoglobin; *MCHC* mean corpuscular hemoglobin concentration; *PLT* platelets; *NEU* neutrophils; *LYM* lymphocytes; *MONO* monocytes; *EOS* eosinophils; *BASO* basophils; *CRP* C reactive protein; *p* level of significance)

Parameters		*Blastocystis* spp.		t-Student test	p
		Positive	Negative		
WBC [tys/µl]	AM ± SD	6.01 ± 1.17	6.13 ± 4.43	0.13	0.90
	Median	6.37	5.64		
	Range	3.81–7.71	2.30–77.27		
RBC [mln/µl]	AM ± SD	4.55 ± 0.27	4.57 ± 0.31	0.24	0.81
	Median	4.48	4.57		
	Range	4.24–5.25	3.77–5.58		
HGB [g/dl]	AM ± SD	13.46 ± 1.08	13.53 ± 1.02	0.39	
	Median	13.50	13.60		0.70
	Range	9.50–15.80	8.90–16.40		
HCT [%]	AM ± SD	39.14 ± 2.76	40.90 ± 27.17	0.32	0.75
	Median	39.40	39.00		
	Range	30.50–46.30	28.20 ± 470.70		
MCV [fl]	AM ± SD	86.12 ± 3.76	85.53 ± 4.73	− 0.61	0.54
	Median	86.70	86.00		
	Range	71.90–92.70	36.50–95.40		
MCH [pg]	AM ± SD	29.60 ± 1.75	29.60 ± 2.05	0.00	1.00
	Median	29.80	30.00		
	Range	22.40–31.80	7.90–33.60		
MCHC [g/dl]	AM ± SD	34.35 ± 0.85	35.32 ± 15.10	0.32	0.75
	Median	34.50	34.60		
	Range	31.10–35.60	30.80–352.00		
PLT [K/µl]	AM ± SD	263.60 ± 48.90	254.30 ± 59.40	− 0.77	0.44
	Median	262.00	246.00		
	Range	175.00–391.00	65.00–630.00		
NEU [K/µl]	AM ± SD	3.28 ± 0.89	3.25 ± .1.16	− 0.15	0.88
	Median	3.23	3.07		
	Range	1.77–4.92	0.97–8.04		
LYM [K/µl]	AM ± SD	2.05 ± 0.65	1.96 ± 0.80	− 0.57	0.57
	Median	1.84	1.88		
	Range	1.41–4.20	0.88–13.70		
MONO [K/µl]	AM ± SD	0.48 ± 0.11	0.46 ± 0.13	− 0.54	0.59
	Median	0.49	0.44		
	Range	0.25–0.72	0.18–0.95		
EOS [K/µl]	AM ± SD	0.16 ± 0.09	0.17 ± 0.29	0.28	0.78
	Median	0.13	0.13		
	Range	0.01–0.37	0.00–5.39		
BASO [K/µl]	AM ± SD	0.03 ± 0.02	0.23 ± 4.06	0.23	0.81
	Median	0.03	0.03		
	Range	0.01–0.08	0.00–85.30		
CRP [mg/l]	AM ± SD	2.19 ± 1.79	2.28 ± 2.71	0.18	0.86
	Median	1.40	1.25		
	Range	1.00–8.38	1.00–34.80		

### *Blastocystis* subtypes and the patients’ characteristics

The characteristics of participants with *Blastocystis* subtypes is presented in Table 4. Three women, one with ST2 and two with ST3 infection did not complete the questionnaire, therefore in Table 4 are presented only 23 participants out of 26 infected with *Blastocystis* spp.

**Table 4:** *Blastocystis* spp. subtype among pre- and perimenopausal women (*BMI* Body Mass Index)

Age	Residence	Tap water drinking	Contact with animals	Travelling abroad	Symptoms	BMI	Chronic diseases
ST1							
58	Urban	Yes	Yes	No	–	21.80	–
46	Rural	Yes	Yes	No	–	28.80	–
65	urban	Yes	Yes	No	Abdominal pain	24.90	Hypertension
46	Urban	Yes	Yes	No	Abdominal pain	27.30	Hypertension
52	Urban	Yes	Yes	No	–	25.30	–
ST2							
61	Urban	Yes	Yes	No	Abdominal pain	20.80	Thyroid disease, coronary artery diseases
50	Urban	Yes	Yes	No	–	31.00	Thyroid disease, autoimmune disease
49	Urban	Yes	Yes	Yes	–	24.60	–
56	Urban	Yes	Yes	Yes	Abdominal pain	36.30	Hypertension, thyroid disease
48	Urban	Yes	Yes	No	Abdominal pain	29.80	-
54	Urban	Yes	Yes	No	Abdominal pain	23.90	Thyroid disease
ST3							
56	Urban	No	Yes	No	–	35.00	Thyroid disease
57	Urban	Yes	Yes	No	–	25.40	–
48	Urban	Yes	Yes	No	–	28.80	–
57	Urban	Yes	Yes	No	Abdominal pain	24.00	–
59	Urban	Yes	Yes	No	–	22.90	–
ST4							
50	Urban	Yes	Yes	No	Abdominal pain	29.00	–
63	Urban	Yes	Yes	No	Abdominal pain	24.60	Hypertension
51	Urban	Yes	Yes	No	–	25.30	–
ST6							
53	Urban	Yes	Yes	No	–	20.00	–
50	Urban	Yes	Yes	No	Abdominal pain	28.80	–
ST7							
45	Urban	Yes	Yes	No	–	32.80	–
ST9							
60	Urban	Yes	Yes	No	–	23.90	–

The mean age of the women infected by ST1 was 53.4 years. Four women lived in the city and one in the countryside. All of them drank tap water, had contact with animals, and did not leave the country. Two women had abdominal pain and hypertension.

The average age of the women with ST2 was 51.57 years. All women were city dwellers, drank tap water, and had contact with domestic animals. Two women traveled to tropical countries. Three women had abdominal pain. Four women were diagnosed with thyroid diseases, with one of them also had high blood pressure and one suffered from coronary artery disease.

The mean age of the women with ST3 was 55.4 years. All women came from the city, had contact with domestic animals, and did not leave the country. 87.7% of the women drank tap water. One woman complained about abdominal pain. One of the women was diagnosed with thyroid disease.

The average age of the women with subtype 4 was 54.67 years. Two women came from the city, one from a village. They all drank tap water, had contact with domestic animals and did not leave the country. Abdominal pain occurred in two women, including one with hypertension.

Subtype 7 was found in a 45-year old woman. She was from a large town, had contact with dogs, drank tap water and never left the country.

Subtype 9 was found in a 60-year old woman from a big city who had contact with dogs, did not drink tap water and did not leave the country.

## Discussion

There is no available data in the literature on the prevalence of *Blastocystis* spp. in pre- and perimenopausal women. But many studies were carried out to examine the prevalence of *Blastocystis* spp. in groups of participants of different age and gender. In Europe, in patients of Scottish Parasite Diagnostic Laboratory, Glasgow (UK) *Blastocystis* spp. infection was detected in 3.9% of subjects using in vitro culture and direct-light microscopy. The infection was most prevalent in patients between 71 and 80 years of age [45]. In cross-sectional study in Spain, *Blastocystis* spp. were detected in 7% of outpatients over 6 years using direct-light microscopy [46]. El-Safadi et al. [47] examined the prevalence of *Blastocystis* spp. in patients of 11 hospitals throughout France (n = 788), who were between 7 months and 95 years (mean age of 45.7 ± 21.3 years), using quantitative PCR. The prevalence of *Blastocystis* spp. was shown to reach 18.1% [47]. A higher prevalence of *Blastocystis* spp. (24.2%) was reported in patients from the Netherlands, including 230 females (median age 29, range 1–82) and 212 males (median age 34, range 0–87 years). The authors used the combined gold standard of sequence-confirmed PCR and positive advanced microscopy [5]. In Poland, the prevalence of *Blastocystis* spp. ranged from 0.89% in patients at the Laboratory of Department of Biology and Medical Parasitology of the Pomeranian Medical University in Szczecin in the years 1983–2012 [48] to about 10% in young people (average age was 23 years) [49] and children in Olsztyn (NE Poland) [50] in 2010. On the other hand, Płonka and Dzbeński [51] did not detect the presence of *Blastocystis* spp. in fecal samples of children aged 7 from various regions of Poland. In the present study *Blastocystis* spp. infection was found in 6.1% of pre- and perimenopausal women. Negative diagnoses are based solely on microscopy. However, the results of our study and those from other authors are difficult to compare due to the selection of the patients and methods used for the diagnosis of *Blastocystis* spp. infection. In addition, there are no data for *Blastocystis* spp. infection in pre- and perimenopausal women.

### *Blastocystis* subtypes

In our study, seven *Blastocystis* subtypes (ST1-ST4, ST6, ST7, and ST9) were identified in pre- and perimenopausal women. Humans in Europe in different age and gender groups are generally infected by *Blastocystis* spp. subtypes 1, 2, 3 and 4 [52–55]. Subtypes 1 and 2 have low specificity and their infections probably result from animal-to-human contact [58]. They are found in humans and a wide range of animals, including dogs, cats, chickens, cattle, pigs, and non-human primates [59–61].

Some researchers suggest that ST1 is the most pathogenic subtype of *Blastocystis* spp. Rene et al. [62] found that ST1 was the most common subtype of *Blastocystis* spp. in patients with diarrhea. Subtype ST1 has been reported from 10.8 to 29% of patients from Poland [13, 56, 63]. In the study presented here, ST1 was found in 19.2% of *Blastocystis* spp. infection in pre- and perimenopausal women.

*Blastocystis* ST2 infections are most often asymptomatic, but gastrointestinal problems and chronic urticaria sometimes happen [64]. In Italy, in mildly symptomatic patients, or those affected by inflammatory bowel disease (IBD), irritable bowel syndrome (IBS), chronic diarrhea, or with different immunosuppression, ST2 represented 14% of the isolates [65]. The prevalence of ST2 infection ranged from 9 to 19% in Poland [46, 56]. In our study, ST2 was found in 26.9% of *Blastocystis* spp. infected pre- and perimenopausal women.

Subtype 3 is classified as anthroponotic and is also found in ungulates, dogs, and non-human primates [61, 66]. Forsell et al. [67] concluded that ST3 was more common in males compared to females. Many researchers suggest that ST3 correlates with gastrointestinal symptoms. *Blastocystis* subtype 3 was detected in the splenic cysts of an immunocompetent woman in Brazil [68]. This subtype of *Blastocystis* spp. is also the most frequently found in patients from Poland; subtype 3 was found in about 30% to 65% of the examined patients [13, 49, 63]. In our study of pre- and perimenopausal women, 26.9% of *Blastocystis* spp. represents ST3.

Subtype 4, common in humans and other primates, has also been found in hoofed mammals, rodents, and birds [54, 66]. ST4 has been associated with infectious diarrhea in European patients [55]. In patients from Denmark with acute diarrhea (lasting more than 2 weeks), ST4 was the most common in Danish *Blastocystis*-positive patients (76%) [55]. Kotłowski [63] found this subtype in Polish travelers to the subtropics and tropics, but also in those who had not left the country. In our study, ST4 was found in 11.5% of *Blastocystis* spp. infections in pre- and perimenopausal women.

Subtypes 5–8, most likely of animal origin, have rarely been observed in humans [69]. ST5 is commonly isolated from livestock and is prevalent in Australia’s pig farmers [70]. ST6 and ST7 from birds are rarely found in Europe, but frequently in humans reported in Japan, Pakistan, and Egypt. In Poland, ST6 has been detected in hens [71]. El Safadi et al. [46] found ST6 and ST7 in 2.1% of isolates from patients in 11 hospitals throughout France. Rene et al. [62] found ST6 in one patient with solid stools. Forsell et al. [67], who studied patients from Stockholm (Sweden), found ST7 (1.6%) in the examined isolates. In the study conducted in central Poland, 8.7% of *Blastocystis* spp. isolates were ST6 and 4.3% were ST7 [56]. In the north of Poland, ST6 and ST7 constituted 3.3% of isolates each [57]. ST6 and ST7 were found in 7.7% of premenopausal and 3.8% of perimenopausal women from the north-west of Poland infected by *Blastocystis* spp. The ST8 subtype has been observed in animals, such as rodents, birds and arthropods, and in some New World monkeys [58, 72]. Single cases of ST8 have been reported in patients from Denmark [62] and Italy [60]. In this study, none of the pre- and perimenopausal women had ST8.

ST9 has only been found in humans and is characterized by multiple intestinal symptoms. Before our study, in which we found cases of this subtype, it had been identified in patients from Denmark [73] and Iran [74].

### High-risk populations

Low socioeconomic status, poor quality of drinking water, consumption of contaminated food and poor personal hygiene habits are important risk factors for intestinal parasitic infection, including *Blastocystis* spp. [75, 76]. Paulos et al. [77] found that *Blastocystis* spp. were detected in urban areas (44%) more often than in rural areas (33%). Similarly, *Blastocystis* spp. were more often detected in residents of urban areas (64.8%) than of rural areas (35.2%) in Northern Cyprus [52]. Lepczyńska et al. [13] also found a higher prevalence of *Blastocystis* spp. in people living in urban areas (68%). In our study, most of *Blastocystis* spp. infected pre- and perimenopausal women came from urban areas.

Contamination of drinking water may be the source of *Blastocystis* spp. infections. It has been shown that *Blastocystis* spp. cysts can survive in chlorinated water at routine concentrations [78]. Leelayoova et al. [2] reported the presence of *Blastocystis* spp. subtype 1 in drinking water. Seyer et al. [52] found no significant correlation between the type of drinking water (bottled or tap water) and the prevalence of *Blastocystis* infection. In our study, more than 90% of pre- and perimenopausal infected with *Blastocystis* spp. drank tap water.

Contact with animals involves a higher risk of *Blastocystis* spp. infection [17, 61]. Zoonotic transmission may be indicated by the high prevalence of *Blastocystis* spp. in animal handlers [55, 61, 66, 79]. Blastocystosis is a potential zoonosis as suggested by a number of studies that have isolated identical STs of *Blastocystis* spp. from humans and the animals they are in contact with [2, 17]. For example, *Blastocystis* spp. infection has been reported in 42% of animals and 63% of zookeepers from the zoo in Western Australia [17]. Some authors suggest the potential role of domestic animals, including dogs and cats, as natural reservoirs of human *Blastocystis* spp. infection. *Blastocystis* spp. have been reported in sheltered canine and feline populations (from ~ 10 to ~ 70%) [80–82]. *Blastocystis* subtypes 2-5 were found in people and domestic dogs living in an urban community in the Philippines [83].

### *Blastocystis* spp. infection and travel to tropical country

Research suggests that the risk of *Blastocystis* spp. infection may increase by traveling to tropical countries due to poor hygiene and sanitation in some areas. Bart et al. [5] observed significantly a higher prevalence of *Blastocystis* spp. infection in patients returning from tropical countries as compared to patients without travel history. Duda et al. [84] found *Blastocystis* spp. in 15.3% of Polish military personnel returning from peacekeeping missions in Iraq and Afghanistan. A prospective, multi-center COMBAT study among Dutch travelers in 2001 [85] showed *Blastocystis* spp. infection in 36.3% of subjects. Among patients from France higher prevalence of *Blastocystis* spp. (27.5%) was in people who had traveled during the last 12 months than in non-travelers (14.7%) [46]. In another study [62], the prevalence of *Blastocystis* spp. among patients with travel-associated diarrhea was significantly higher than among patients with chronic diarrhea. In patients who traveled to tropical countries from Poland, the prevalence of ST2 (22.8%) was significantly higher than in those who did not leave the country (10%) [57]. In our study, pre- and perimenopausal women who had recently been in a tropical country had ST2. However, Stensvold et al. [35] did not find any correlation between the subtypes of *Blastocystis* spp. and travel activity up to three months before stool sampling.

### Symptoms and *Blastocystis* spp. infection

The pathogenic role of *Blastocystis* spp. in causing clinical symptoms remains a controversial issue due to the morphology and genetic diversity of protozoa, as well as the different immune responses of the host [86]. It is usually associated with nonspecific gastrointestinal symptoms, including abdominal pain, diarrhea, nausea, vomiting, bloating, anorexia, and, less commonly, urticaria, intense itching [64, 87], and iron deficiency anemia [88–90].

Some researchers have observed *Blastocystis* spp. infections more frequently in patients with intestinal symptoms than in asymptomatic patients [91, 92]. In Mexico, Diaz et al. [93] found *Blastocystis* spp. in 7% of children with diarrhea. The prevalence of *Blastocystis* spp. in Thai patients with diarrhea was 21% [94]. In our study, 43.5% of pre- and perimenopausal women suffered from gastrointestinal symptoms (including abdominal pain), although we found no relationship between *Blastocystis* spp. infection and gastrointestinal symptoms. Similarly, Seyer et al. [52] did not notice a statistically significant correlation between *Blastocystis* spp. infection and gastrointestinal symptoms, such as bloating, abdominal pain, abdominal cramps, constipation, diarrhea, and nausea.

The feces of *Blastocystis* spp. infected patients contain cysts or vacuolar forms. The ameboid form is probably pathogenic and is mostly associated with showing by the patient some symptoms [95]. A large number of parasites in the intestine (> 5 parasites per high-power field) also entails gastrointestinal symptoms [3]. In this study, all pre- and perimenopausal women infected with *Blastocystis* spp. had less than five vacuolar forms per high-power field.

Many studies have been devoted to the correlation between different *Blastocystis* spp. subtypes and their pathogenic potential. Kaneda et al. [96] suggested that STs 1, 2, and 4 might be responsible for gastrointestinal symptoms. Yan et al. [97] only found ST1 in a group of symptomatic patients. In their study of Spanish patients with acute and chronic diarrhea, Domínguez-Márquez et al. [53] observed ST4, ST2 and ST1 in 94.1%, 3.9% and 2% of the participants, respectively. Mattiucci et al. [65] observed ST3 (46%), ST4 (21.7%), ST1 (15.3%), ST2 (13.8%), ST6 (3.2%) and ST8 (0.5%) in *Blastocystis* spp. infected mildly symptomatic patients with IBD, IBS, chronic diarrhea, or another immunosuppression. In this study, gastrointestinal symptoms were observed in pre- and perimenopausal women infected by ST3 and ST4 (both 66.7%), ST6 (50%) and ST1 (40%).

### *Blastocystis* spp. infection and laboratory results of blood

The pathomechanism of *Blastocystis* spp. infection is still unknown. Yavasoglu et al. [88] observed a significantly higher rate of *Blastocystis* spp. infection among patients with iron deficiency anemia (IDA), and so did El Deeb et al. [90] in their study of pregnant women. Another study by El Deeb et al. [98] showed a significantly higher prevalence of *Blastocystis* spp. in patients with anemia compared to non-anemic patients. Cheng et al. [99] observed lower hemoglobin, hematocrit and neutrophil counts in patients with *Blastocystis* spp. infection. Some studies explained anemia in infected patients with the fact that cationic ferritin is essential for the endocytic pathway of *Blastocystis* spp. [100]. However, in our study, similar to Chen et al. [101], we did not find any significant differences in the hematological parameters between *Blastocystis* spp. infected and uninfected patients. In *Blastocystis* spp. infected group of patients, only one woman had lower hemoglobin, hematocrit, and MCV parameters which suggest microcytic anemia. Laodim et al. [94] found that *Blastocystis* spp. infected patients had lower peripheral eosinophils and serum albumin levels. Levy et al. [102] and Nassir et al. [30] also reported that *Blastocystis* spp. infection is associated with hypoalbuminemia. In the histological examination of the cecum and colon of *Blastocystis* spp. infected mice, intense inflammatory-cell infiltration, edematous lamina propria and mucosal sloughing were observed [103]. It is suggested that *Blastocystis* spp. are able to change leukocyte levels, and induce the production of inflammatory cytokines [102, 103]. In our study, there was no difference between white blood cells and CRP levels between *Blastocystis* spp. infected and uninfected pre- and perimenopausal women. Among *Blastocystis* spp. infected patients, only one woman who was infected with ST2 had a higher level of CRP. The exact relationship between the hematological and biochemical parameters of blood and blastocystosis needs further analysis.

### *Blastocystis* spp. infection and chronic diseases

Immunocompromised patients, including those with AIDS/HIV infection, myeloproliferative disorders, and treated with chemotherapy, are more frequently infected with *Blastocystis* spp. [104]. *Blastocystis* spp. have been observed in 38% of patients with AIDS or HIV infection, as found at the Infectious Diseases Department of a tertiary referral university hospital in northern Germany [11] *Blastocystis* spp. infection (subtypes 3, 4, 6, and 7) was observed in 16% of patients aged around 55 with hematological malignancies (HM; n = 94) in France [105]. Stensvold et al. [55] found *Blastocystis* spp. in 23% of AIDS/HIV infected patients from Denmark, including ST3 in 11.5%, ST1 in 9.4%, ST1 and ST3 in 2.1%.

A higher prevalence of *Blastocystis* spp. has been observed in patients with IBS compared to healthy populations or patients with other gastrointestinal disorders. Studies of IBS patients showed a higher prevalence of ST1, ST3, and ST4 isolates of *Blastocystis* spp. and ST7 was found to be correlated with IBS. Karasartova et al. [106] detected *Blastocystis* spp. in 30% of splenectomized patients.

In this study, 57.1% of pre- and perimenopausal women with ST2 suffered from a thyroid disease (Hashimoto’s disease). Rajič et al. [107] provided evidence that the eradication of *Blastocystis* spp. can prevent the development of both symptomatic Hashimoto’s thyroiditis and chronic urticaria.

This work has certain limitations. We acknowledge that the screening tests were conducted using direct-light microscopy and the *CoproELISA Blastocystis* test which are characterized by low sensitivity. That’s why we cannot assure that the prevalence of *Blastocystis* spp. in this study was not higher. However, the overall results can be compared with studies using the same test method. There is no control group. Nevertheless, the analysis of *Blastocystis* spp. subtype allowed us to demonstrate the population structure of *Blastocystis* spp. and reveal the presence of ST9 in pre- and perimenopausal women. However, the pathogenicity and risk factors of *Blastocystis* spp. infection in pre- and perimenopausal women are not fully understood and require further research.

## Conclusions

Our study provides new insights into the epidemiology of *Blastocystis* spp. and the prevalence and ST distribution of the parasite in Poland. Moreover, this research complements the limited available data on the prevalence of *Blastocystis* spp. in pre- and perimenopausal women.

The study is the first report showing the presence of ST9 in Poland. Our results showed no association between *Blastocystis* spp. infection and gastrointestinal symptoms as well as biochemical and hematological blood tests. Taking into account specific risk factors, we found that pre- and perimenopausal women who lived in urban regions or drinking tap water have a bigger chance of getting infected with *Blastocystis* spp. However, further research in this area is needed.

## Data Availability

The data of this manuscript is available at https://ppm.pum.edu.pl/info/researchdata/PUMec675cc289d448ab9824f6c86805eb87/
